# Localized Vulvar Micro-lymphatic Imbalance Mimicking Recurrent Labia Minora Hypertrophy After Labiaplasty: Clinicopathologic Correlation and Mechanistic Insights

**DOI:** 10.7759/cureus.104893

**Published:** 2026-03-09

**Authors:** Pablo González-Isaza, Manuel Sánchez-Prieto, Rafael Sánchez-Borrego

**Affiliations:** 1 Department of Urogynecology, Hospital Universitario San Jorge, Pereira, COL; 2 Department of Obstetrics and Gynecology, Instituto Universitario USP Dexeus, Barcelona, ESP; 3 Department of Obstetrics and Gynecology, DIATROS, Clínica de Atención a la Mujer, Barcelona, ESP

**Keywords:** labia minora hypertrophy, labiaplasty, lymphatic anatomy, surgical revision, vulvar lymphedema

## Abstract

Persistent focal enlargement after labiaplasty is commonly attributed to aesthetic under-resection or postoperative scar remodeling; however, disruption of superficial vulvar lymphatic pathways has not been well characterized as a potential contributor to postoperative contour abnormalities. We describe a clinicopathologic case of localized lymphatic channel dilation presenting as apparent recurrent labia minora hypertrophy following primary labiaplasty and propose an anatomically grounded, hypothesis-generating framework. A 16-year-old patient developed unilateral apical enlargement 90 days after neurovascular-preserving CO_2_ laser labiaplasty, prompting revision surgery. Excised tissue underwent histopathologic evaluation, with findings interpreted in the context of contemporary vulvar lymphatic mapping data. Intraoperative assessment demonstrated focal subepithelial fibrosis and stromal thickening confined to the apical labial segment. Histologic analysis revealed multiple dilated, thin-walled lymphatic channels within edematous fibrotic stroma, without evidence of mesenchymal neoplasia. The topographic distribution corresponded to the superior superficial vulvar lymphatic pathway described in indocyanine green mapping studies. These findings suggest that localized micro-lymphatic imbalance may represent a rare but anatomically plausible mechanism underlying select cases of apparent recurrence after labiaplasty. Recognition of superficial vulvar drainage pathways may refine interpretation of postoperative asymmetry and inform surgical technique in anatomically concentrated regions. Prospective studies incorporating lymphatic imaging and histologic validation are warranted.

## Introduction

Labiaplasty has become one of the most frequently performed procedures in aesthetic genital surgery, with contemporary series reporting high patient satisfaction and low complication rates [1,2]. Nevertheless, revision surgery remains necessary in a subset of patients, with published estimates ranging from approximately 5% to 19% depending on technique and indication [2,3]. Persistent or recurrent labial enlargement is commonly attributed to aesthetic under-resection, contour asymmetry, scar remodeling, or patient dissatisfaction [2,3]. However, the biological mechanisms underlying focal postoperative enlargement have not been systematically examined.

The labia minora are not merely cutaneous folds but complex mucocutaneous structures containing dense microvascular, neural, and lymphatic networks. Histopathologic analyses of labia minora hypertrophy have consistently demonstrated lymphangiectasia and stromal edema, findings interpreted by some authors as features of localized chronic lymphedema or microvascular remodeling [4,5]. Whether these structural features represent a primary lymphatic abnormality, a reactive phenomenon, or a compensated drainage state remains unclear.

Recent advances in indocyanine green (ICG) lymphography have delineated reproducible superficial vulvar lymphatic pathways, including a superior pathway coursing along the clitoral hood-frenular complex [4]. This anatomically concentrated region corresponds to the apical segment frequently manipulated during neurovascular-preserving labiaplasty [2,3]. The relationship between superficial lymphatic architecture and postoperative contour changes has not been explored in aesthetic genital surgery.

In other anatomical regions, even limited disruption of superficial lymphatic collectors may alter interstitial fluid dynamics and contribute to localized fibrotic remodeling without producing diffuse edema [4]. Given the compact organization of vulvar lymphatic networks, it is biologically plausible that focal surgical perturbation could produce a regional drainage imbalance that mimics recurrent hypertrophy rather than classical lymphedema [4,6,7].

We report a case of histologically confirmed localized lymphatic channel dilation presenting as apparent recurrence following primary labiaplasty. By integrating clinicopathologic findings with contemporary lymphatic mapping data, we propose a hypothesis-generating anatomical framework that may refine the interpretation of postoperative asymmetry and inform surgical technique.

## Case presentation

A 16-year-old nulligravid patient presented for evaluation of postoperative asymmetry following primary labiaplasty. Her gynecologic history was otherwise unremarkable, with regular menstrual cycles since menarche and no history of vulvovaginal infections, pelvic inflammatory disease, or prior genital procedures. She reported no previous symptoms of vulvar swelling, asymmetry, or discomfort before the primary surgery. Her past medical history was negative for chronic disease, connective tissue disorders, or lymphatic conditions. She had no previous surgical history apart from the primary labiaplasty, and there was no known family history of lymphedema, vascular malformations, or hereditary connective tissue disorders.

**Figure 1:**
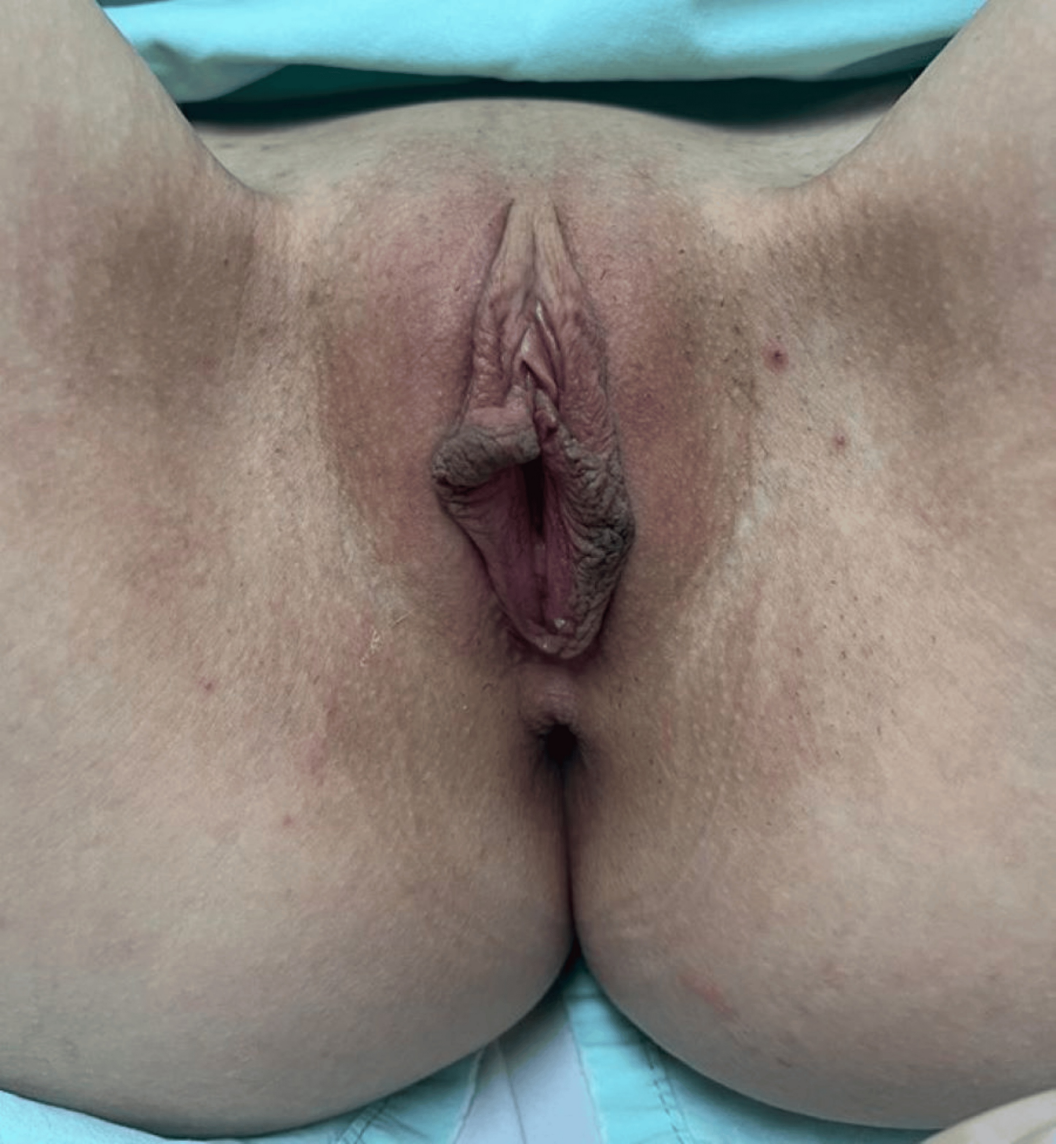
Preoperative Anatomic Configuration Preoperative clinical photograph demonstrating bilateral horizontal bifurcation of the labia minora with apical projection and complex topographic configuration of the clitoral hood-frenular complex. No asymmetry or focal enlargement was present at baseline.

Prior to the primary procedure, the patient presented with bilateral labia minora hypertrophy associated with aesthetic concern and occasional mechanical discomfort with tight clothing. Physical examination demonstrated elongated labial tissue with a complex apical configuration, including bilateral horizontal bifurcation of the free labial edge. No focal enlargement, edema, induration, or inflammatory signs were observed at baseline, and the surrounding vulvar tissues appeared otherwise normal. The preoperative anatomic configuration is shown in Figure 1.

Primary procedure

A neurovascular-preserving topographic labiaplasty was performed using a pulsed CO_2_ laser (4 W, 8 Hz, HP mode, 380 μm spot size). Resection included the apical labial complex while preserving clitoral neurovascular structures. Hemostasis was achieved with controlled laser coagulation. Closure was tension-free. The postoperative course was uncomplicated.

Postoperative evolution

**Figure 2:**
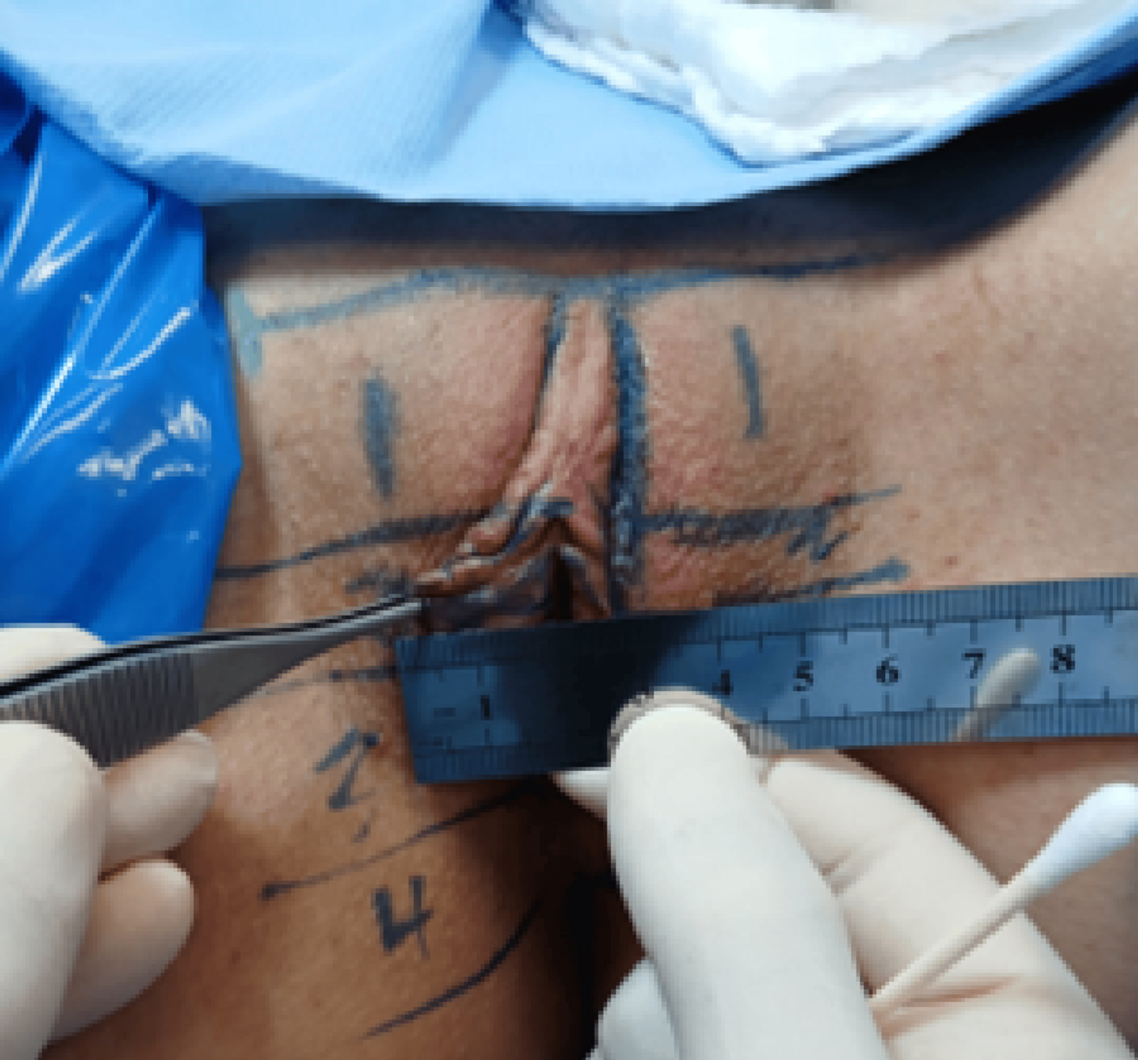
Unilateral Apical Enlargement at 90 Days After Primary Labiaplasty Clinical photographs obtained at 90 days postoperatively demonstrating focal enlargement of the right labia minora localized to the clitoral frenulum-apical region. The enlargement is confined to the superior labial segment without evidence of diffuse edema or inflammatory changes. The contralateral side preserves a symmetric contour.

At 90 days postoperatively, unilateral enlargement of the right labia minora was observed, localized to the clitoral frenulum-apical region (Figure 2). The tissue was firm, non-tender, and without erythema or fluctuance. The contralateral side maintained the expected contour.

Because the enlargement persisted beyond the expected inflammatory phase and demonstrated focal asymmetry, revision surgery was undertaken.

Revision surgery and intraoperative findings

Revision surgery was performed under radiofrequency-assisted dissection. Intraoperative examination revealed dense subepithelial fibrosis and focal stromal thickening localized to the apical segment. The tissue was firm and partially adherent to adjacent folds. No hematoma, seroma, cystic lesion, or residual mucocutaneous redundancy was identified.

The fibrotic tissue was carefully excised while preserving mucocutaneous continuity and vascular integrity. The distribution and consistency of the affected tissue were not characteristic of simple under-resection but suggested localized structural remodeling.

**Figure 3:**
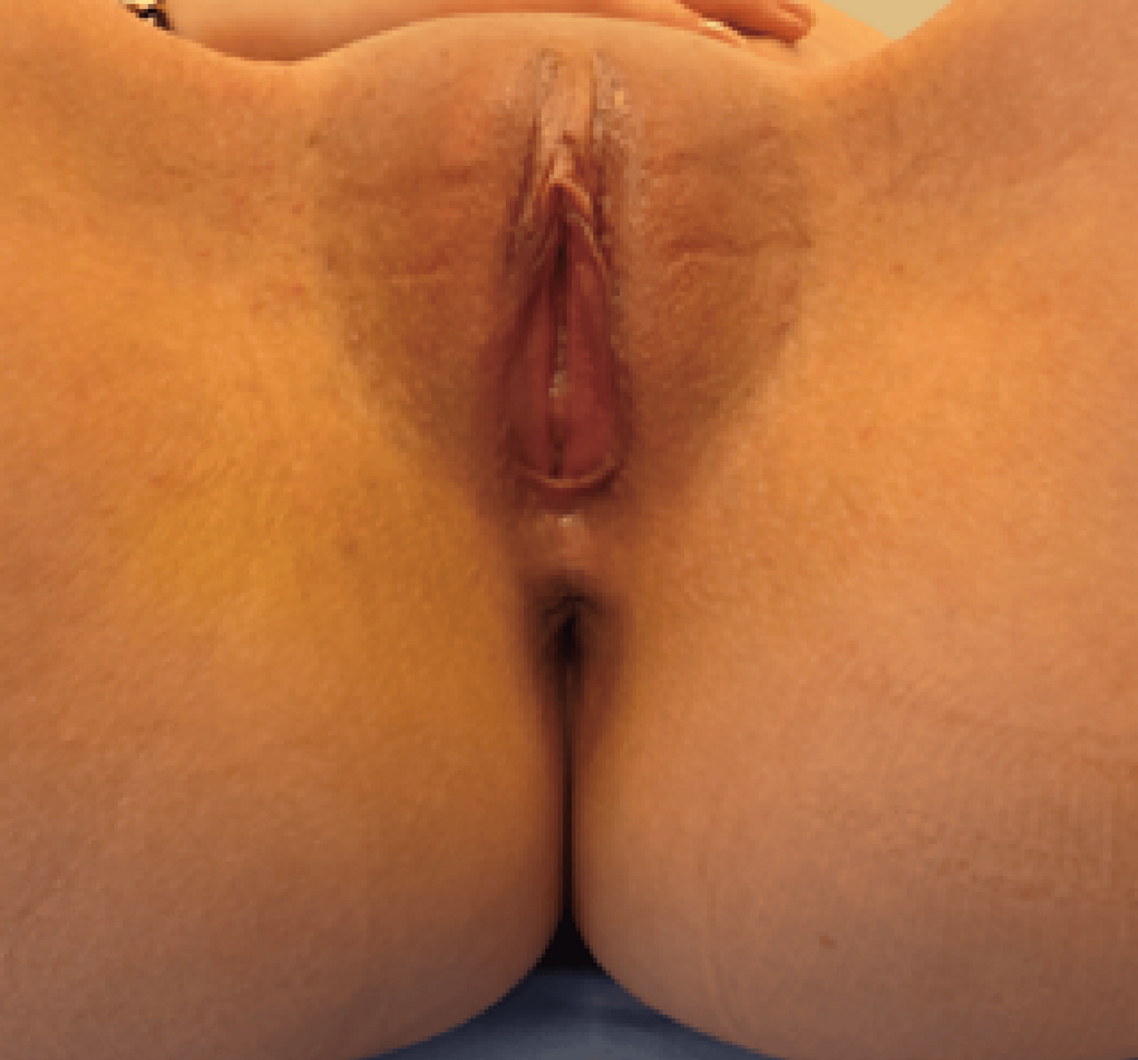
Post-revision Clinical Appearance of the Right Labial Segment Clinical photograph obtained following revision surgery demonstrating restoration of the symmetric apical contour of the right labia minora. The previously observed focal enlargement is no longer evident. Tissue consistency was normalized on clinical examination, and no signs of inflammation or recurrent asymmetry were present at follow-up.

Postoperative healing was uneventful. At follow-up, a symmetric apical contour was restored without evidence of recurrent enlargement or inflammatory changes (Figure 3).

Histopathologic findings

**Figure 4:**
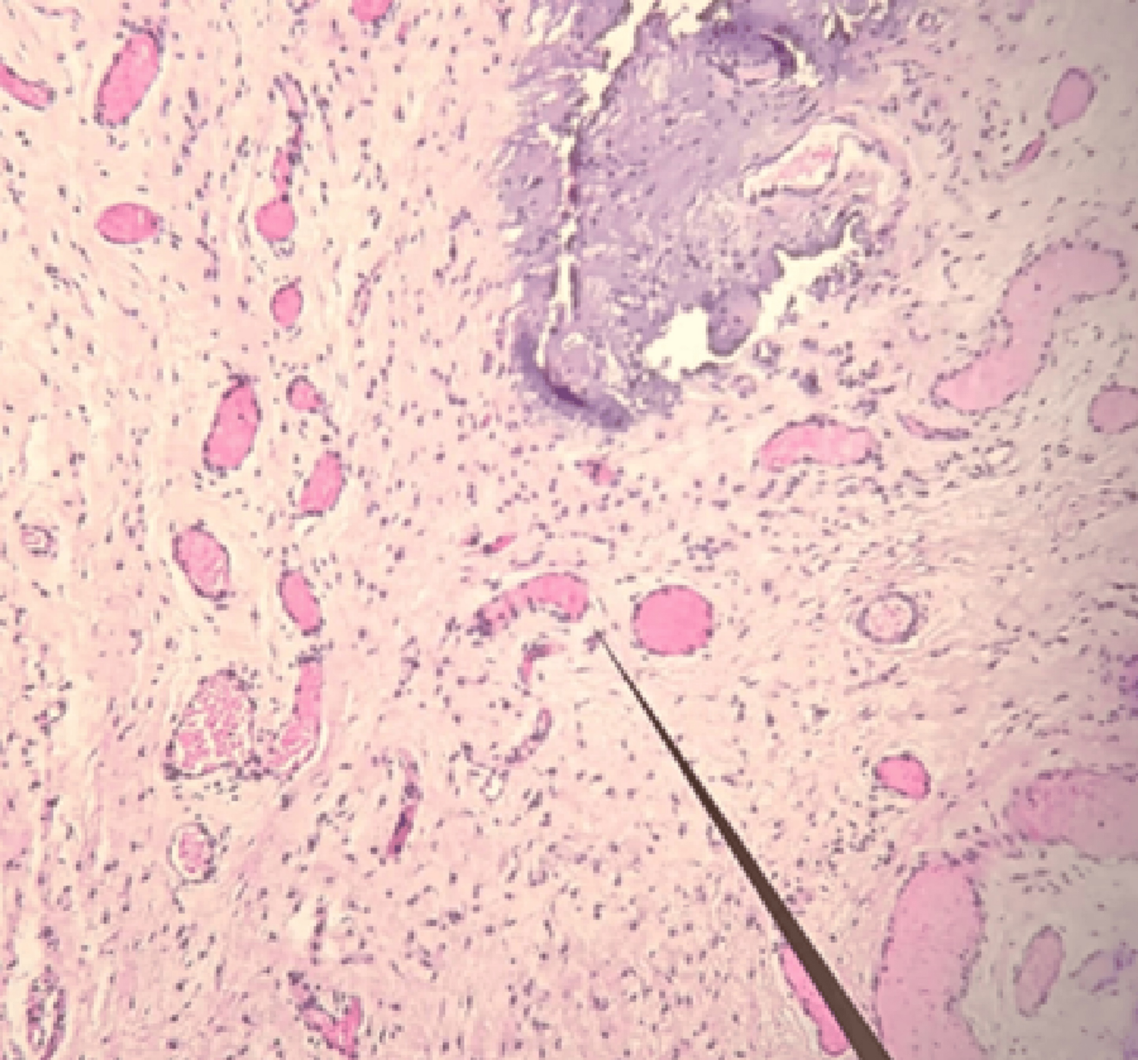
Histopathologic Findings of the Resected Apical Labial Tissue (hematoxylin-eosin stain, original magnification ×200) Representative hematoxylin and eosin-stained section of excised apical labial tissue. The image demonstrates expanded subepithelial stroma with increased collagen deposition and scattered spindle-shaped fibroblasts consistent with fibrotic remodeling. Multiple dilated, thin-walled vascular channels are identified within the stroma (arrow), lacking a prominent muscular layer and containing minimal intraluminal cellular elements. Adjacent small-caliber vessels with erythrocyte-filled lumina are also present. No cytologic atypia, necrosis, or features suggestive of mesenchymal neoplasia are observed.

Microscopic examination demonstrated multiple dilated lymphatic channels within loose edematous stroma, accompanied by subepithelial fibrosis, findings compatible with localized lymphatic dilation within fibrotic stroma (Figure 4) [5,6]. Periodic acid-Schiff (PAS) staining confirmed the presence of lymphatic spaces embedded within fibrotic tissue.

The distribution of dilated lymphatic channels extended beyond patterns typically seen in isolated scar remodeling, suggesting the presence of a microvascular component beyond isolated fibrotic remodeling. No myxoid stromal proliferation, vascular wall thickening, or infiltrative growth pattern was observed, excluding aggressive angiomyxoma and related mesenchymal lesions [7].

Vulvar lymphatic vulnerability hypothesis

To contextualize this observation, we propose a staged, hypothesis-generating framework integrating established principles of secondary lymphedema pathophysiology with contemporary vulvar lymphatic mapping. Histopathologic analyses of labia minora hypertrophy have demonstrated lymphangiectasia and stromal edema [5]. These findings may represent a compensated micro-lymphatic dilation state rather than clinically manifest lymphedema [5]. In such a scenario, superficial drainage remains functionally sufficient despite structural enlargement of lymphatic channels.

ICG lymphography has delineated three superficial vulvar lymphatic pathways, including a superior pathway coursing along the clitoral hood-frenular complex [4]. This anatomically concentrated region corresponds to the apical labial segment frequently manipulated during topographic labiaplasty, providing structural plausibility for localized drainage perturbation [4].

We therefore propose the vulvar lymphatic vulnerability hypothesis as a staged, hypothesis-generating framework integrating baseline micro-lymphatic architecture, surgical perturbation, fibrotic remodeling, and clinical expression (Table 1).

**Table 1:** Proposed Staged Framework of Vulvar Lymphatic Vulnerability Conceptual, hypothesis-generating framework integrating baseline micro-lymphatic architecture, surgical disruption, and fibrotic remodeling to explain focal postoperative enlargement. The model does not establish causation but provides an anatomically coherent explanation consistent with histologic findings and contemporary superficial vulvar lymphatic mapping.

Stage	Structural Condition	Pathophysiologic Change	Clinical Correlate
Baseline (Compensated)	Mild micro-lymphatic dilation within organized superficial drainage pathways	Preserved regional flow despite structural enlargement of lymphatic channels	No clinical enlargement
Surgical Perturbation	Mechanical or thermal disruption of superficial collectors, particularly in apical drainage territories	Altered regional lymphatic flow dynamics without diffuse edema	Subclinical regional imbalance
Fibrotic Remodeling	Progressive subepithelial fibrosis and increased interstitial resistance	Reduced microvascular drainage efficiency	Localized interstitial fluid accumulation
Clinical Expression	Persistent focal overload within an anatomically concentrated territory	Stromal induration and fluid retention	Firm, asymmetric enlargement mimicking recurrent hypertrophy

In this model, a compensated baseline state may include mild micro-lymphatic dilation without clinical manifestation. Surgical manipulation, particularly mechanical or thermal disruption of superficial collectors within anatomically concentrated apical territories, may alter regional flow dynamics without producing diffuse edema. Progressive fibrotic remodeling may subsequently increase interstitial resistance, impairing microvascular drainage efficiency and shifting a previously compensated system toward localized overload. Clinically, this process may manifest as focal fluid accumulation and stromal induration presenting as firm, asymmetric enlargement that mimics recurrent hypertrophy rather than classical lymphedema.

This framework does not establish causation but provides an anatomically and physiologically coherent explanation reconciling histologic findings with contemporary lymphatic mapping. It suggests that, in rare instances, postoperative contour abnormalities may reflect localized micro-lymphatic imbalance rather than isolated aesthetic under-correction.

## Discussion

Persistent focal enlargement following labiaplasty is most attributed to aesthetic under-resection or postoperative scar remodeling [2,3,8]. In the present case, however, the coexistence of dense subepithelial fibrosis and dilated lymphatic channels suggests that postoperative contour abnormalities may, in select circumstances, involve mechanisms extending beyond residual tissue volume or isolated scar hypertrophy.

Table 2 outlines a structured differential framework for postoperative labial enlargement. Aesthetic under-resection typically preserves native tissue characteristics, whereas scar remodeling is characterized by collagen deposition localized to incision planes [2,3]. By contrast, the current case demonstrated dilated, thin-walled lymphatic channels embedded within edematous fibrotic stroma. This histologic pattern exceeds what is generally expected in isolated scar hypertrophy and supports consideration of a localized microvascular component [5,6].

**Table 2:** Differential Mechanistic Framework for Postoperative Labial Enlargement

Feature	Aesthetic Under-Resection	Scar Remodeling/Fibrosis	Proposed Localized Lymphatic Mechanism
Timing of Presentation	Immediate or early postoperative	Weeks to months	Delayed (after initial healing phase)
Distribution	Usually symmetric or proportional	Focal, often along the incision line	Focal, anatomically clustered (apical region)
Tissue Consistency	Soft, like native tissue	Firm, fibrotic	Firm with subepithelial induration
Histological Findings	Normal mucocutaneous structure	Dense collagen deposition, minimal lymphatic dilation	Dilated lymphatic channels within edematous stroma with associated fibrosis
Microvascular Component	Not implicated	Secondary to scar formation	Primary micro-lymphatic dilation contributing to interstitial imbalance
Topographic Correlation	No specific vascular pattern	Along the surgical incision	Corresponds to superficial vulvar lymphatic pathway (clitoral hood-frenular complex)
Response to Revision	Additional resection sufficient	Excision of fibrotic tissue	Excision may relieve localized drainage imbalance
Pathophysiologic Analogy	Residual tissue volume	Hypertrophic scar	Early-stage localized secondary lymphedema

​​​​​Histopathologic analyses of labia minora hypertrophy have reported lymphangiectasia and stromal edema [6], findings that may reflect a compensated micro-lymphatic architecture rather than established lymphatic insufficiency. While structural dilation does not equate to clinical dysfunction, it may denote a baseline state predisposed to secondary perturbation [6].

Contemporary ICG lymphographic studies have demonstrated organized superficial vulvar drainage pathways, including a superior collector trajectory along the clitoral hood-frenular complex [4]. Given the compact organization of these networks, even limited disruption may alter regional flow dynamics. In anatomically concentrated territories, localized imbalance may occur without diffuse edema, potentially manifesting as focal enlargement rather than generalized swelling [4].

In the present case, the preoperative configuration demonstrated bilateral horizontal bifurcation with increased apical topographic complexity. Although no causal relationship can be inferred from a single observation, such morphologic variants may theoretically concentrate superficial lymphatic pathways within a confined drainage territory [4,8]. In anatomically complex apical configurations, disruption of superficial collectors could have a disproportionate regional impact [4]. Whether specific labial morphologic patterns predispose patients to postoperative micro-lymphatic imbalance warrants systematic investigation.

On this basis, we propose the vulvar lymphatic vulnerability hypothesis as a staged, hypothesis-generating model integrating baseline micro-lymphatic architecture, surgical perturbation, progressive fibrotic remodeling, and focal clinical expression (Table 1). A previously compensated system may transition toward localized overload following the disruption of superficial collectors combined with increased interstitial resistance.

This framework does not imply that labiaplasty commonly induces lymphatic dysfunction. Rather, it provides an anatomically coherent explanation for rare cases in which postoperative asymmetry cannot be fully accounted for by under-resection or isolated scar remodeling.

Recognition of superficial vulvar drainage pathways and apical anatomical complexity may refine the interpretation of delayed focal enlargement. Careful dissection in anatomically concentrated regions and avoidance of excessive subdermal thermal injury may theoretically reduce disruption of superficial collectors [9-12]. Persistent asymmetry beyond the expected inflammatory phase warrants systematic evaluation before attributing findings solely to aesthetic under-correction.

This report is limited by its single-case design, absence of pre- and postoperative lymphatic imaging, and lack of lymphatic-specific immunohistochemical confirmation. Causality cannot be established, and incidence cannot be inferred. The proposed framework should therefore be considered hypothesis-generating.

Prospective investigations incorporating lymphatic mapping, quantitative volumetric assessment, and immunohistochemical validation are required to determine whether localized micro-lymphatic imbalance represents an underrecognized contributor to revision labiaplasty.

## Conclusions

This case describes localized lymphatic channel dilation presenting as apparent recurrence after labiaplasty. When interpreted alongside contemporary vulvar lymphatic mapping, the findings support the plausibility of a localized micro-lymphatic imbalance mechanism in anatomically concentrated apical regions. The proposed vulvar lymphatic vulnerability hypothesis provides a conceptual framework for understanding select cases of postoperative labial enlargement beyond aesthetic under-resection or isolated scar remodeling. Prospective studies incorporating lymphatic imaging and histologic validation are required to determine the incidence and clinical relevance of this mechanism.

Localized postoperative enlargement after labiaplasty is not always attributable to aesthetic under-resection or scar remodeling. In rare cases, localized micro-lymphatic imbalance may contribute to focal enlargement mimicking recurrent hypertrophy. Awareness of superficial vulvar lymphatic pathways may aid interpretation of postoperative asymmetry and guide surgical technique in anatomically concentrated apical regions.
